# Performance of urinary neutrophil gelatinase-associated lipocalin, clusterin, and cystatin C in predicting diabetic kidney disease and diabetic microalbuminuria: a consecutive cohort study

**DOI:** 10.1186/s12882-017-0620-8

**Published:** 2017-07-12

**Authors:** Xian-Fei Zeng, Dong-Xue Lu, Jun-Min Li, Yun Tan, Zhuo Li, Lei Zhou, Zeng-Qian Xi, Shu-Miao Zhang, Wei Duan

**Affiliations:** 1 Department of Laboratory Medicine and Blood Transfusion, Shaanxi Corps Hospital, Chinese People’s Armed Police Forces, Xi’an, 710054 China; 2 Department of Anesthesiology, Xijing Hospital, Fourth Military Medical University, Xi’an, 710032 China; 3 Department of Laboratory Medicine, The First Affiliated Hospital, Xi’an Medical University, Xi’an, 710077 China; 4 Department of Laboratory Medicine, Xijing Hospital, Fourth Military Medical University, Xi’an, 710032 China; 5 Department of Cardiology, Shaanxi Corps Hospital, Chinese People’s Armed Police Forces, Xi’an, 710054 China; 6 Department of Endocrinology, Shaanxi Corps Hospital, Chinese People’s Armed Police Forces, Xi’an, 710054 China

**Keywords:** Clusterin, Cystatin C, Diabetic kidney disease, Diagnostic biomarker, Neutrophil gelatinase-associated Lipocalin

## Abstract

**Background:**

Tubular biomarkers have been regarded as emerging and promising markers for early diagnosis of diabetic kidney disease (DKD). The study was to determine the diagnostic capabilities of tubular biomarkers (urinary neutrophil gelatinase-associated lipocalin [NGAL], clusterin, and cystatin C) for DKD and diabetic microalbuminuria, and whether or not the tubular biomarkers appear earlier than microalbuminuria.

**Methods:**

In this consecutive cohort study, 146 type 2 diabetes mellitus (T2DM) patients with a disease duration of ≥6 years were enrolled. Thirty age- and gender-matched subjects without any systemic diseases were recruited as the control group. Urinary samples collected before treatment were tested for NGAL, clusterin, and cystatin C.

**Results:**

The levels of biomarkers were higher in patients with DKD (*p* < 0.001); and positively correlated with the urinary albumin creatinine ratio (UACR; *p* < 0.001). With respect to the diagnosis of DKD, the areas under the receiver operating characteristic curve (AUCs) for urinary NGAL, clusterin, and cystatin C were 0.816 (95% confidence interval [CI], 0.741–0.891), 0.775 (95% CI: 0.694–0.857), and 0.803 (95% CI: 0.722–0.884), respectively. The levels of urinary NGAL and cystatin C in the normoalbuminuria group (UACR <30 mg /g•Cr) were elevated compared with the control group, unlike urinary clusterin. There was no statistical difference in the levels of the three biomarkers between groups with different levels of haemoglobin A_1C_ (HbA_1c_). The diagnostic AUCs for urinary NGAL, clusterin, and cystatin C in patients with diabetic microalbuminuria were 0.841 (95% CI: 0.775–0.907), 0.783(95% CI: 0.710–0.856), and 0.805 (95% CI: 0.733–0.877), respectively.

**Conclusions:**

Urinary NGAL, clusterin, and cystatin C may be promising biomarkers for diagnosing DKD and diabetic microalbuminuria. It is possible that urinary NGAL and cystatin C increase before the onset of microalbuminuria in T2DM patients.

## Background

Diabetic kidney disease (DKD) resulting from systemic microvascular impairment is traditionally referred to as diabetic nephropathy (DN), remains the leading cause for end-stage renal disease, and is a significant risk for cardiovascular morbidity and mortality [1, 2]. Within the diabetic kidney, the glomeruli and tubules are subject to damage from hyperglycemia, advanced glycosylation products, activation of inflammatory cytokines, and microalbuminuria, which ultimately develop into renal fibrosis with renal failure [3, 4]. Once an irreversible lesion occurs in the diabetic kidney, it is unlikely that renal function can recover. Understanding the precise pathogenesis underlying DKD and exploring novel biomarkers for early diagnosis play crucial roles in developing more effective preventive and therapeutic strategies [5].

At present, the diagnosis of DKD mainly relies on the Kidney Disease Outcome Quality Initiative (KDIGO) guidelines, the standards of which include duration of diabetes (6–10 years) and an increase in the threshold value of the urinary albumin excretion rate [6]. The utility of the two KDIGO criteria cannot effectively realize the goal for the early diagnosis of DKD. The rate of awareness regarding the duration of diabetes in new cases is not high, especially in less developed areas. Proteinuria, a hallmark of renal injury, has insufficient power to predict and categorize DKD due to low sensitivity and specificity [7]. A large proportion of renal impairment in diabetes patients is associated with non-proteinuria [8, 9]. The predictive value and existing threshold for diagnosing abnormal levels of albuminuria have come under question [10, 11]. Hence, there is a critical need to identify reliable biomarkers for individuals who are at risk for developing DKD that maybe progress to end-stage renal disease [7].

A growing body of evidence indicates that some novel tubular biomarkers have excellent predictability for chronic kidney disease (CKD) or acute kidney injury (AKI), and may exhibit a promising perspective on the early diagnosis for DKD, such as neutrophil gelatinase-associated lipocalin (NGAL), cystatin C (CysC), and clusterin (CLU) [12–16]. NGAL, a glycoprotein belonging to the lipocalin superfamily, is one of the most commonly studied novel biomarkers for renal impairment. NGAL can be expressed in various cells. The stimuli that induce epithelial damage lead to NGAL high expression and then increase the baseline serum level. NGAL filtered by the glomerulus will be captured by proximal tubules and only a minimal amount is excreted in the urine. Tubular injury results from ischemia, inflammation, and hyperglycemia leads to a decrease in NGAL reabsorption and an increase in NGAL secretion by tubular cells [17]. CysC is produced in all nucleated cells, and is a 13.4-kDa cysteine protease inhibitor. CysC is freely filtered by glomeruli and is completely taken up by proximal tubular cells without tubular secretion. CysC is not normally present in the urine in significant amounts. Fluctuation in the urinary CysC level is mainly due to decreased reabsorption from injured/dysfunctional tubules [18]. CLU is a 75 kD-disulfide-linked heterodimeric glycoprotein with multiple biologic functions involving sperm maturation, lipid transportation, complement inhibition, tissue remodeling, and membrane recycling. CLU protein is highly expressed by the TGF-β signaling pathway in renal tubular epithelia after renal injury and can be deposited in the kidney as a component of immune deposits [19]. Thus, it can be assumed that the level of each marker in urine may change significantly, given that tubular impairment plays an important role in the pathogenesis underlying DKD [20, 21]. Previous studies have demonstrated that markedly increased levels of biomarkers are positively correlated with albuminuria and reflect the severity of renal damage in diabetic cohorts [22–24] or animal models [13, 25, 26]. Renal impairment that is mainly caused by hyperglycemia, however, has a different and more complicated pathogenesis compared with renal ischemia, hypoxic damage, or toxic injury, and thus the feasibility and efficacy of these biomarkers warrants further confirmation in clinical studies. To date, the clinically comprehensive performance of novel biomarkers has rarely been reported for the diagnosis of DKD and diabetic albuminuria. Moreover, it is unclear whether or not these markers have sufficient changes for clinical detection before the onset of microalbuminuria.

The goal of this study was to evaluate the diagnostic capacity of urinary NGAL, CLU, and CysC for DKD and diabetic albuminuria in T2DM patients. Furthermore, the temporal profile of the three biomarkers was determined to illustrate the potentially additional superiority in the diagnosis of DKD and diabetic albuminuria.

## Methods

### Study design

T2DM patients with a documented disease duration of ≥6 years and who did not receive any treatment during the previous 2 weeks before admission to Shaanxi Province Corps Hospital of the Chinese People’s Armed Police Forces (Xi’an, China) between June and October 2015 were eligible for enrollment in this observational cohort study. T2DM was defined according to the American Diabetic Association criteria (2014). The exclusion criteria included chronic infections, malignancy, immunologic disorders, hypertension or use of anti-hypertension medications, severe liver dysfunction, a recent (within 6 months) history of acute myocardial infarction or stroke, urinary tract infection, primary glomerulonephritis, hypertensive nephropathy, lupus nephritis, interstitial nephritis or prior kidney transplantation. Patients were diagnosed with DKD and non-DKD in accordance with the DKD standard of KDIGO [6]. DKD was defined as a decrease in the estimated glomerular filtration rate (eGFR) to 60 mL/min/1.73 m^2^, as estimated by the Modification of Diet in Renal Disease formula (eGFR [mL/min/1.73 m^2^] = 186.3 × [serum creatinine]^-1.154^× [age]^-0.203^ × 0.742 if female), or the presence of albuminuria, including microalbuminuria (UACR 30–299 mg/g•Cr) and macroalbuminuria (UACR ≥ 300 mg/g) [27, 28]. Further, all recruited patients were divided into 3 groups based on the level of the urinary albumin creatinine ratio (UACR; mg albumin/g creatinine) as follows: normoalbuminuria (UACR <30 mg /g•Cr); microalbuminuria (UACR 30–300 mg /g•Cr); and overt nephropathy (UACR >300 mg/g•Cr and/or persistent proteinuria). Additionally, 30 respective age- and gender-matched subjects without any systemic diseases, such as diabetes, hypertension, cardiovascular disease, or renal insufficiency, were chosen as the control group. Complete medical records, including demographic characteristics, duration of diabetes, renal function, records of physical examinations, and results of laboratory testing, were documented on all subjects. Fasting serum and urine samples were collected in non-heparinized tubes and centrifuged at 1000×*g* at 4 °C for 5 min within 1 h of collection. Then, the supernatants were stored at −80 °C until the biomarker assay was performed. The study protocol was approved by the Institution Review Board of the Shaanxi Province Corps Hospital of the Chinese People’s Armed Police Forces and written informed consent was obtained from all patients.

### Serum and urinary analyses

Urinary NGAL and CLU were measured in duplicate by means of commercial ELISA (R&D System, Minneapolis, MN, USA) according to the manufacturer’s instructions. The detection limits of the assays for NGAL and CLU were 0.04 and 1.05 ng/mL, respectively. The intra- and inter-assay coefficients of variation (CVs) were <4.5% and <8.0% at NGAL concentrations from 1.14–7.54 and 1.05–6.92 ng/ml, respectively. The intra- and inter-assay CVs were <3.7% and <8.4% at CLU concentrations from 19.0–130.0 and 22.1–136 ng/mL, respectively. Urinary microalbumin, CysC, and high-sensitivity C-reactive protein were measured using an immunoturbidimetric method (Cobas c501; Roche Diagnostics, Mannheim, Germany). Glycosylated hemoglobin (HbA_1c_) in whole blood was quantitatively detected using a D-10 Hemoglobin Testing System (Bio-Rad, Schiltigheim, France) and ion exchange chromatography. The remaining related laboratory parameters, including serum and urinary creatinine, serum cholesterol, triglycerides, and glucose, were obtained using common biochemical detection by standard operating procedures. UACR was calculated by comparing the albumin concentration in the urinary sample against the creatinine concentration (mg/g•Cr).

### Statistical analysis

Statistical analyses were performed with SPSS (version 19.0; SPSS, Inc., Chicago, IL, USA) and GraphPad Prism 5 (GraphPad Software, Inc., La Jolla, CA, USA) for Windows. A *p*-value <0.05 was considered statistically significant. Continuous variables were presented as the mean ± SD or median (25th and 75th percentiles) and categorical data were expressed as percentages. Any testing value below the detection limit was assumed to be the detection limit. Student’s *t*-test, the Mann–Whitney *U*-test, or the Kruskal–Wallis test, as appropriate, was used to compare the means or medians of continuous variables between or among groups. Categorical data were tested using a χ2-test. Spearman correlation coefficients were calculated to determine the relationships between biomarkers. Biomarkers were identified as independent factors or not associated with DKD by multivariate logistic analysis. The diagnostic test characteristics for DKD, macroalbuminuria, and microalbuminuria were determined by the area under the curve (AUC)-receiver operating characteristic (ROC) analysis. A non-parametric method [29] was used to test the differences in ROC curves. The biomarker cut-off values for diagnosis were acquired based on the best Youden index.

## Results

### Characteristics of the study cohort

A total of 146 T2DM patients were recruited into the cohort, and consisted of 42 DKD and 104 non-DKD patients. The characteristics of the DKD, non-DKD, and control groups are described in Table 1. With respect to age, gender composition, blood pressure, serum cholesterol, serum triglycerides, and body mass index, there were no significantly differences among the three groups. Compared with the subjects diagnosed with DKD, non-DKD patients had higher levels of HbA_1c_, a shorter duration of diabetes, a lower serum creatinine level, a lower frequency of diabetic retinopathy and a higher eGFR. There was a significant elevation in urine microalbuminuria and UACR in the DKD group as compared with non-DKD patients (*p* = 0.000). The novel biomarkers, including urinary NGAL, CLU, and CysC were significantly increased in DKD patients (*p* = 0.000).

**Table 1:** Patient demographic data and clinical characteristics

Characteristics	DKD(*n* = 42)	non-DKD(*n* = 104)	Control(*n* = 30)	*p* value
Age (years)^a^	55.7 ± 15.7	57.6 ± 12.7	55.9 ± 13.3	0.815
Male (%), n	57.1% (24)	57.7% (60)	56.7%(17)	0.920
Diabetes duration (years)^a^	14.0 ± 3.6	9.3 ± 2.3	**-**	**0.000**
Systolic pressure (mmHg)^a^	119.5 ± 8.4	116.5 ± 10.7	117.3 ± 9.2	0.113
Diastolic pressure (mmHg)^a^	77.6 ± 5.7	77.3 ± 4.1	78.7 ± 5.0	0.789
HbA_1c_ (%)^a^	7.8 ± 2.3	8.5 ± 1.8	5.2 ± 0.9	**0.015**
BMI (kg/m^2^) ^a^	24.5 ± 0.84	23.4 ± 0.67	23.0 ± 0.75	0.734
hs-CRP (mg/l) ^b^	8.2 (4.6, 10.7)	10.1 (5.6, 15.8)	1.8 (0.8, 2.8)	**0.000**
Diabetic retinopathy (%), n	35.5% (15)	19.2% (20)	**-**	**0.035**
Serum creatinine (mg/dl)^b^	1.49 (1.18,1.84)	0.86 (0.76,1.02)	0.92 (0.81, 1.12)	**0.000**
eGFR (ml/min per 1.73m^2^)^b^	78.0 (68.5, 89.4)	93.5 (89.0, 97.3)	92.3 (85.6, 94.3)	**0.000**
Serum cholesterol (mg/dl)^a^	177.7 ± 40.8	188.4 ± 41.4	183.5 ± 38.9	0.213
Serum triglycerides (mg/dl)^a^	202.9 ± 82.1	189.1 ± 81.1	162.0 ± 65.6	0.278
Urine microalbuminuria (μg/ml)^b^	285.0 (60.1, 514.6)	16.7 (7.1, 37.6)	11.3 (5.5, 14.3)	**0.000**
UACR (mg/g•cr)^b^	405.0 (90.6, 752.7)	24.7 (13.1, 57.1)	26.6 (12.5, 37.7)	**0.000**
Urine NGAL (ng/ml)^b^	113.0 (37.5, 549.8)	26.0 (15.8, 58.3)	13.2 (11.0, 16.0)	**0.000**
Urine CLU (ng/ml)^b^	761.0 (388.7, 986.3)	253.4 (136.5, 572.6)	228.4 (157.3, 301.7)	**0.000**
Urine CysC (ng/ml)^b^	0.85 (0.56, 1.30)	0.30 (0.19, 0.44)	0.18 (0.12, 0.19)	**0.000**

*DKD* diabetic kidney disease, *HbA*_1c_ haemoglobin A_1C_, *BMI* body mass index, *hs-CRP* high-sensitivity C-reactive protein, *eGFR* estimated glomerular filtration rate, *UACR* urinary albumin creatinine ratio, *NGAL* neutrophil gelatinase-associated lipocalin, *CLU* clusterin, *CysC* Cystatin C. ^a^ mean ± SD, ^b^ median (IQR). *p* values in bold numerals suggest significant values.

### Correlation analysis between biomarkers

Table 2 presents the correlations between two biomarkers related to the UACR, eGFR, urinary NGAL (uNGAL), urinary CLU (uCLU), and urinary CysC (uCysC). Positive correlations were detected between UACR and each biomarker, as follows: UACR and uNGAL (*r* = 0.563, *p* = 0.000); UACR and uCLU (*r* = 0.549, *p* = 0.000); and UACR and uCysC (*r* = 0.594, *p* = 0.000). Negative correlations were detected between eGFR and each biomarker. Additionally, there was a positive correlation between uNGAL and uCLU, uNGAL and uCysC, and uCLU and uCysC (*p* = 0.000). Multivariate logistic analysis showed that the biomarkers were independent factors associated with DKD (uNGAL, odds ratio [OR]: 2.010 [95% CI: 1.064–4.016], *p* = 0.001; uCLU, OR: 1.010 [95% CI: 1.004–1.016], *p* = 0.034; uCysC, OR: 2.826 [95% CI: 1.073–7.441], *p* = 0.035).

**Table 2:** Correlation studies of UACR, eGFR, uNGAL, uCLU and uCysC in T2DM patients (*n* = 146)

	uNGAL		uCLU		uCysC	
	r	*p*	r	*p*	r	*p*
UACR	0.563	0.000	0.549	0.000	0.594	0.000
eGFR	−0.654	0.000	−0.489	0.000	−0.533	0.000
uNGAL	-	-	0.428	0.000	0.413	0.000
uCLU	0.428	0.000	-	-	0.415	0.000
uCysC	0.413	0.000	0.415	0.000	-	-

*UACR* urinary albumin creatinine ratio, *eGFR* estimated glomerular filtration rate, *uNGAL* urinary neutrophil gelatinase-associated lipocalin, *uCLU* urinary clusterin, *uCysC* urinary Cystatin C, *T*2*DM* type 2 diabetic mellitus, *r* Spearman correlation test

### Biomarkers levels at different degrees of renal injury

**Fig. 1:**
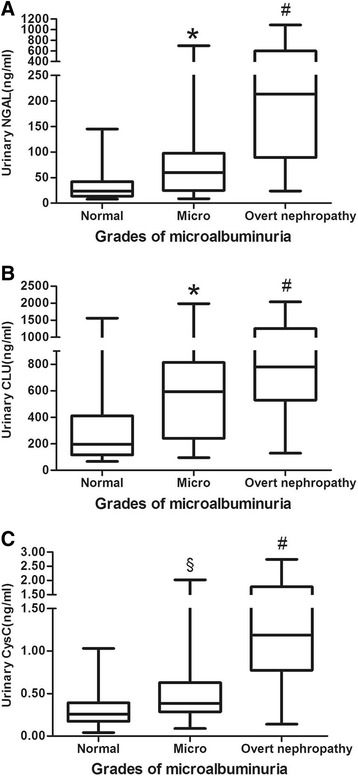
The levels of biomarkers in different grades of microalbuminuria. **a** urinary NGAL at different grades of microalbuminuria; **b** urinary CLU at different grades of microalbuminuria; **c** urinary CysC at different grades of microalbuminuria. ^*^ *p* < 0.001 compared with grade Normal; ^#^ *p* < 0.001 compared with grade Micro; ^§^ *p* < 0.05 compared with grade Normal. NGAL, neutrophil gelatinase-associated lipocalin; CLU, clusterin; CysC, cystatin C; Normal, normoalbuminuria; Micro, microalbuminuria

All T2DM patients were divided into three groups based on the level of microalbuminuria (Fig. 1). The uNGAL of T2DM patients with overt nephropathy was higher than patients with microalbuminuria (*p* = 0.000) and normoalbuminuria (*p* = 0.000). The microalbuminuria group also had an elevated uNGAL level compared with the normoalbuminuria group (*p* = 0.000). The same trends were present with respect to the uCLU and uCysC levels. With the increase in the level of urinary microalbumin, all three urinary biomarkers levels were significantly elevated in T2DM patients.

### Diagnostic performance of urinary biomarkers for DKD or macroalbuminuria

**Fig. 2:**
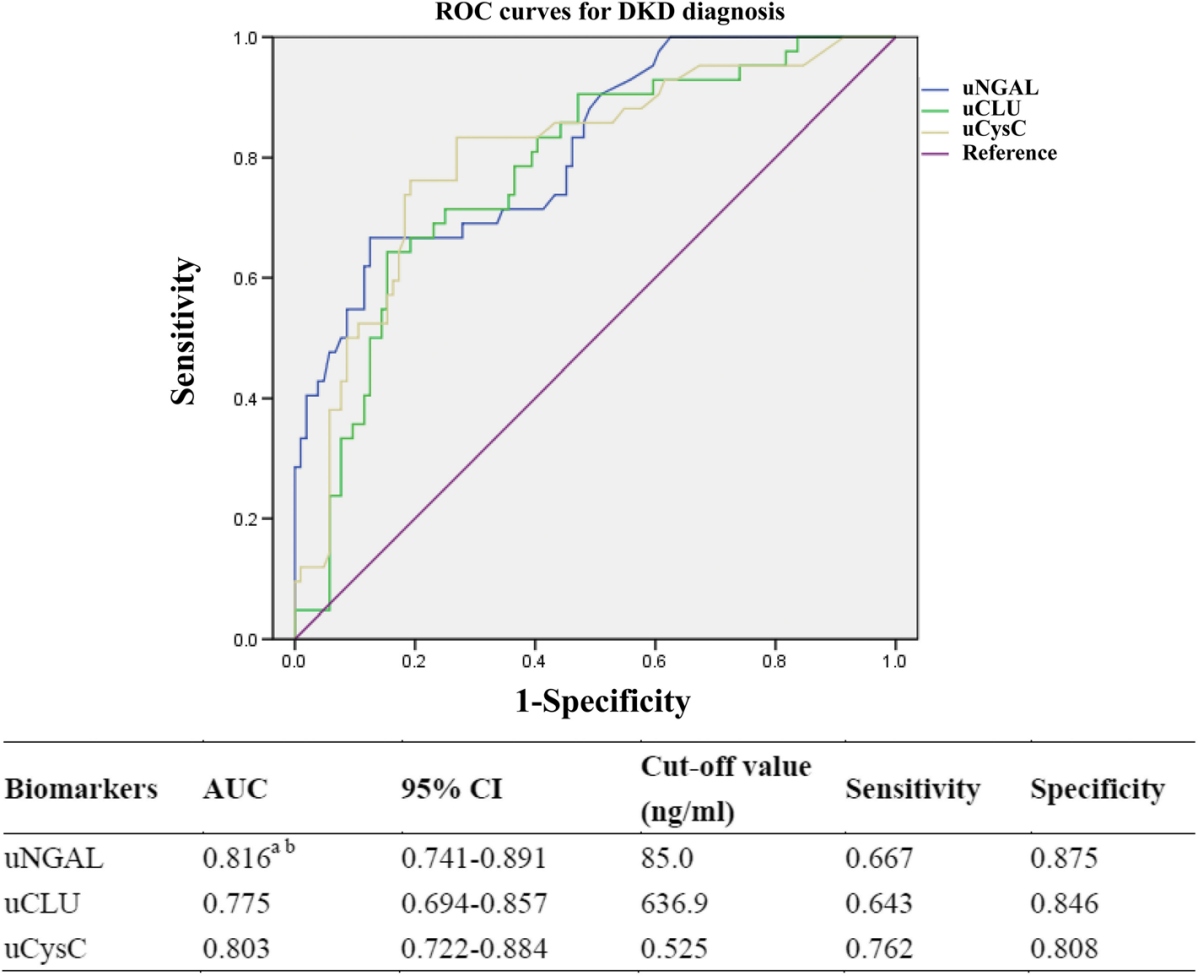
ROC of biomarkers for DKD diagnosis. The AUC and the cut-off value of each urinary biomarker are presented in the separate table under the figure. ^a^Compared with uCLU, *p* < 0.05; ^b^Compared with uCysC, *p* > 0.05. DKD, diabetic kidney disease; AUC, area under the receiver operating characteristic curve; CI, confidence interval; uNGAL, urinary neutrophil gelatinase-associated lipocalin; uCLU, urinary clusterin; uCysC, urinary cystatin C

**Fig. 3:**
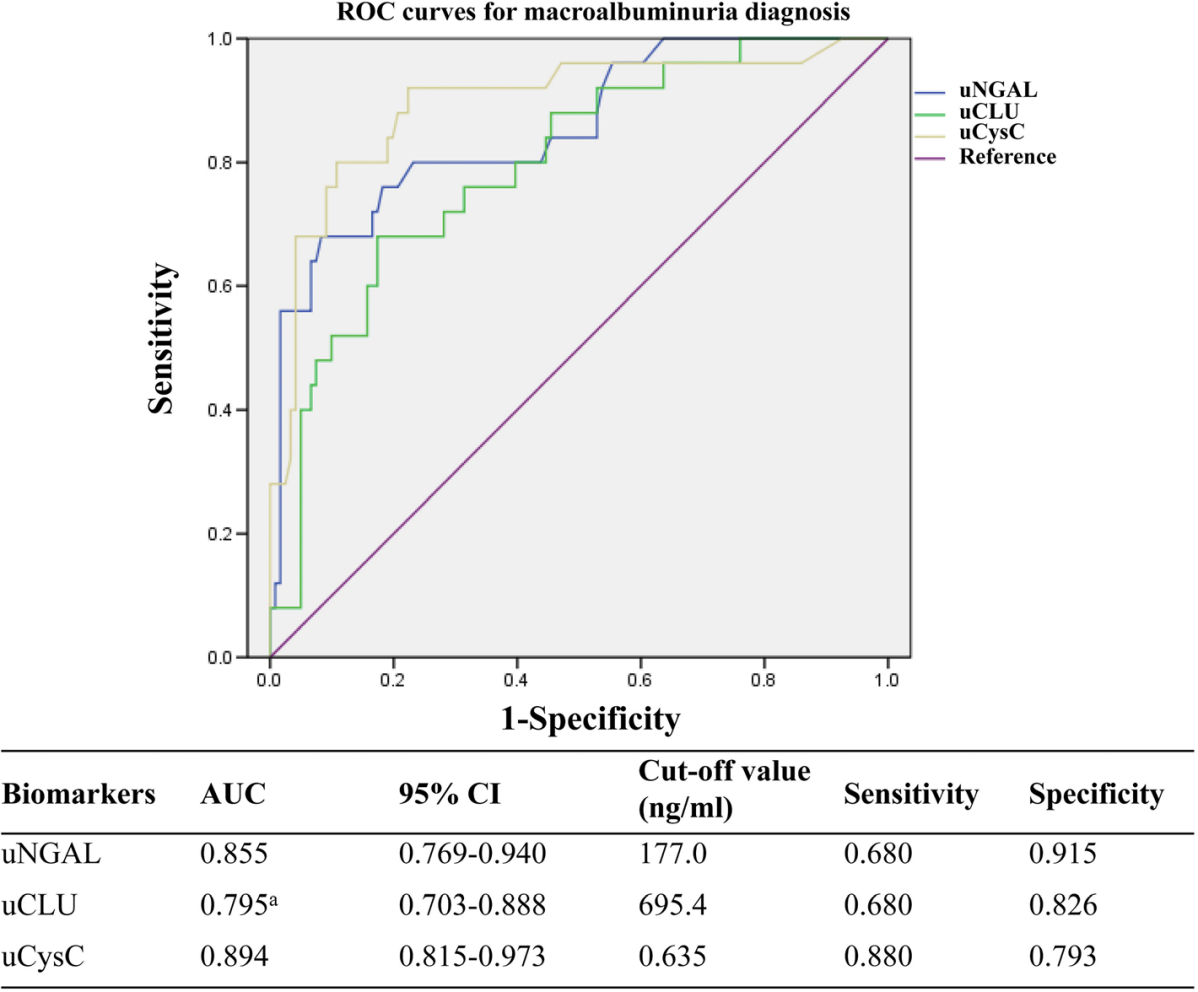
ROC of biomarkers for macroalbuminuria diagnosis. The AUC and the cut-off value of each urinary biomarker are presented in the separate table under the figure. ^a^Compared with uNGAL, *p* < 0.05. AUC, area under the receiver operating characteristic curve; CI, confidence interval; uNGAL, urinary neutrophil gelatinase-associated lipocalin; uCLU, urinary clusterin; uCysC, urinary cystatin C

Figure 2 presents the ROC curves of novel urinary biomarkers for distinguishing DKD from T2DM patients. The AUCs for the diagnosis of DKD using uNGAL, uCLU, and uCysC were 0.816 (95% CI: 0.741–0.891), 0.775 (95% CI: 0.694–0.857), and 0.803 (95% CI: 0.722–0.884), respectively. At the corresponding optimal cut-off values, the sensitivities and specificities of these biomarkers to predict DKD reached 66.7% and 87.5%, 64.3% and 84.6%, and 76.2% and 80.8%, respectively. Of these novel biomarkers, uNGAL and uCysC had larger AUCs for diagnosing DKD compared to uCLU (*p* < 0.05). The AUCs of the 3 biomarkers (uNGAL, uCLU, and uCysC) for macroalbuminuria were 0.855 (95% CI: 0.769–0.940), 0.795 (95% CI: 0.703–0.888), and 0.894 (95% CI: 0.814–0.973), respectively (Fig. 3).

### Temporal patterns of biomarker appearance in the urine

We determined the temporal characteristics of novel biomarkers at detectable concentrations in patients with chronic renal impairment resulting from T2DM. The focus was whether or not the novel biomarkers appeared earlier than microalbumin in the urine of T2DM patients. The data displayed in Table 3 revealed that the levels of uNGAL and uCysC were increased in both groups with an UACR <30 mg/g•Cr compared with the concentrations in the control group. When the UACR level was in the reference range, these two biomarkers may have changed in the urine; however, uCLU was not significantly different between the control and T2DM or DKD groups with an UACR <30 mg/g•Cr. In addition, between the T2DM and DKD patients with a HbA_1c_ < 7.0% and ≥7.0%, there was no statistical difference in the levels of the 3 biomarkers (Table 4). The higher HbA_1c_ level may not directly result in a change in the urinary biomarkers.

**Table 3:** Urinary biomarkers in control subjects, T2DM and DKD patients with normoalbuminuria

	Control Subjects (*n* = 30)	T2DM patients with UACR <30 mg/g•cr (*n* = 64)	DKD patients with UACR <30 mg/g•cr (*n* = 12)
uNGAL (ng/ml)	13.2 (11.0, 16.0)	19.5 (13.0, 41.0)*	40.5 (36.5, 69.5) **
uCLU (ng/ml)	228.4 (157.3, 301.7)	194.3 (132.5, 398.5)	243.1 (142.3, 372.1)^#^
uCysC (ng/ml)	0.18 (0.12, 0.19)	0.24 (0.17, 0.36)^§^	0.50 (0.36, 0.76) ^§§^
eGFR (ml/min per 1.73m^2^)	92.3 (85.6, 94.3)	90.1 (85.1, 96.2)	53.0 (49.0, 55.3)^¶^

*T2DM* type 2 diabetic mellitus; *DKD* diabetic kidney disease; *UACR* urinary albumin creatinine ratio; *uNGAL* urinary neutrophil gelatinase-associated lipocalin; *uCLU* urinary clusterin; *uCysC* urinary Cystatin C. *p = 0.000 compared with Health Subjects, **p = 0.002 compared with T2DM patients with UACR < 30 mg/g•cr, ^#^ *p* = 0.556 compared among groups, ^§^ *p* = 0.000 compared with Health Subjects, ^§§^ *p* = 0.000 compared with T2DM patients with UACR < 30 mg/g•cr, ^¶^ *p* = 0.000 compared among groups

**Table 4:** Urinary biomarkers of T2DM and DKD patients with different HbA_1c_ levels

	T2DM or DKD patients with HbA_1c_ < 7.0% (*n* = 44)	T2DM or DKD patients with HbA_1c_ ≥ 7.0% (*n* = 104)	*p* value
uNGAL (ng/ml)	48.5 (22.5, 91.8)	37.5 (19.3, 89.8)	0.279
uCLU (ng/ml)	434.3 (140.1, 858.5)	358.2 (185.2, 662.3)	0.925
uCysC (ng/ml)	0.37 (0.22, 1.02)	0.35 (0.23, 0.68)	0.528

*T*2*DM* type 2 diabetic mellitus, *DKD* diabetic kidney disease, *HbA*_1c_ haemoglobin A_1C_, *uNGAL* urinary neutrophil gelatinase-associated lipocalin, *uCLU* urinary clusterin, *uCysC* urinary Cystatin C

### Diagnostic profile of urinary biomarkers for microalbuminuria

**Fig. 4:**
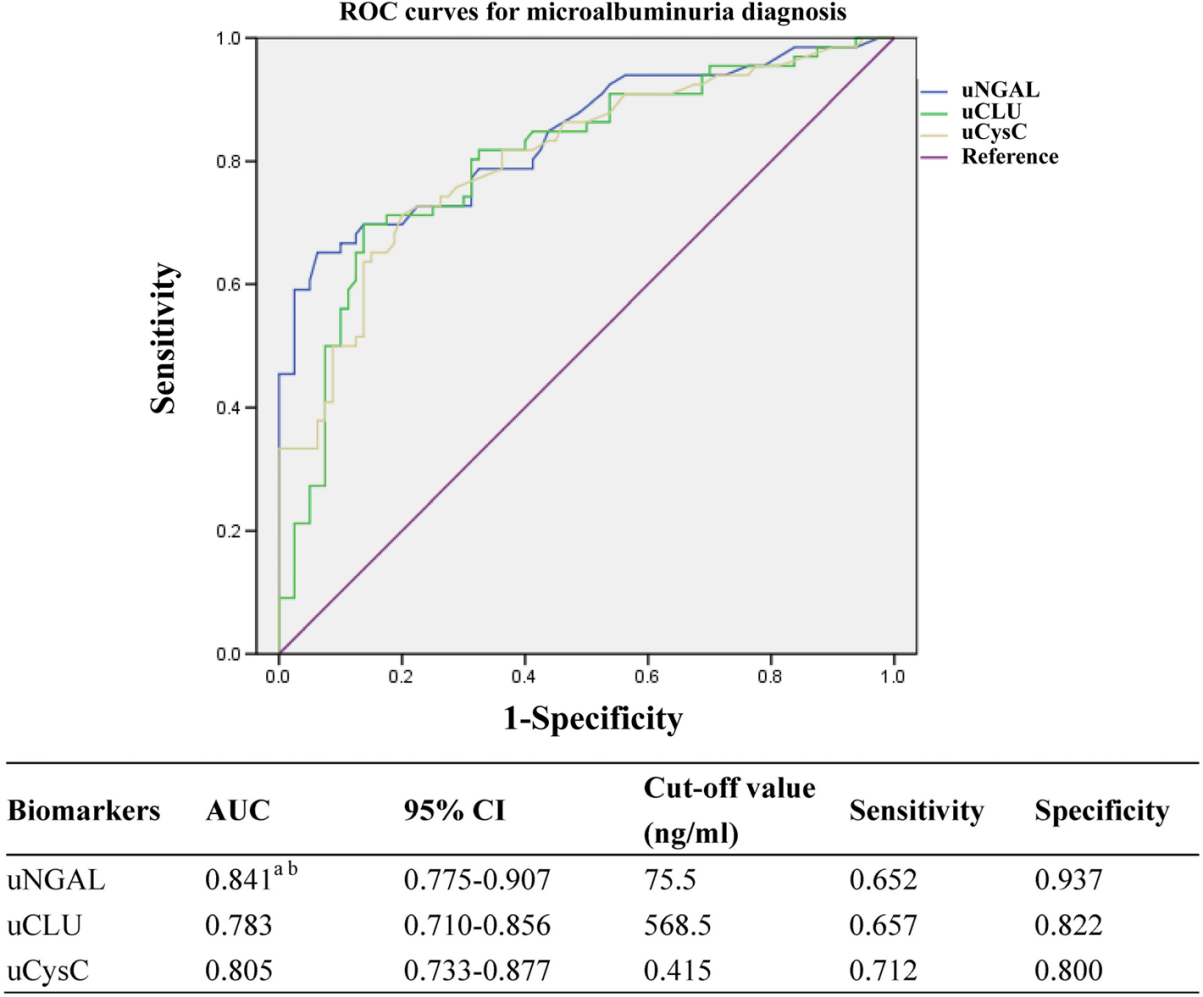
ROC of biomarkers for microalbuminuria diagnosis. The AUC and the cut-off value of each urinary biomarker are presented in the separate table under the figure. ^a^Compared with uCLU, *p* < 0.05; ^b^Compared with uCysC, *p* < 0.05. AUC, area under the receiver operating characteristic curve; CI, confidence interval; uNGAL, urinary neutrophil gelatinase-associated lipocalin; uCLU, urinary clusterin; uCysC, urinary cystatin C

The chronologic difference in the appearance of biomarkers in the urine makes predicting microalbuminuria by uNGAL, uCLU, and uCysC possible and meaningful. An ROC analysis of biomarkers for microalbuminuria in T2DM and DKD patients, as shown in Fig. 4, demonstrated that the AUCs were 0.841 (95% CI: 0.775–0.907), 0.783 (95% CI: 0.710–0.856), and 0.805 (95% CI: 0.733–0.877) for uNGAL, uCLU, and uCysC, respectively. uNGAL and uCysC had larger AUCs compared with the AUC of uCLU (*p* < 0.05), with sensitivities of 65.2% and 71.2%, and specificities of 93.7% and 80.0%, at the corresponding cut-off values 75.5 ng/ml and 0.415 ng/ml, respectively. However, the uCLU with the smallest AUC was predictive of microalbuminuria with a sensitivity of 69.7% and a specificity of 86.2% at the optimal cut-off value of 568.5 ng/ml.

## Discussion

Tubular impairment plays an important role in the pathogenesis underlying DKD [20, 21]. Microalbuminuria activates renal proximal tubular epithelial cells to induce tubulointerstitial inflammation. In contrast, high glucose levels and diabetic substrates, including advanced glycation end-products, carbonyl intermediates, and ultra-filtered growth factors, trigger a number of signaling pathways to promote tubular cell hypertrophy and the interstitial deposition of chemokines, cytokines, growth factors, and adhesion molecules, which are capable of accelerating further inflammation and fibrosis. The extent of tubulointerstitial injury may ultimately determine the attrition rate of renal function in DKD patients [20, 30]. Therefore, tubular biomarkers may be crucial as glomerular markers for early diagnosis and stratification of DKD or renal impairment in T2DM.

Previous studies have demonstrated that urinary NGAL, CLU, and CysC are promising biomarkers for tubular injuries in CKDs [14, 15], AKIs [12, 31], or nephrotoxic lesions [32], specifically reflecting proximal and distal, distal, and proximal tubular injuries, respectively; however, the diagnostic performance for renal impairment in T2DM and the temporal characteristics of appearance in the urine have rarely been investigated in clinical subjects. The results of the present study provide new evidence supporting the potential role of novel biomarkers as candidates for DKD diagnostic tools. The values of biomarkers were higher in the DKD group than the non-DKD group (Table 1), all of which were positively correlated with the current golden criteria for UACR (Table 2) and as independent factors for DKD. The levels of biomarkers were significantly increased in patients with renal insults and normoalbuminuria, microalbuminuria, and overt nephropathy (Fig. 1). ROC curve analyses affirmed that candidates exhibited moderate diagnostic performance for DKD and macroalbuminuria, of which uNGAL and uCysC performed better (AUC = 0.816 and 0.803 for DKD, respectively; AUC = 0.855 and 0.894 for macroalbuminuria, respectively; Figs. 2 and 3). In a study comprised of 70 patients with diabetes, the AUC of urinary NGAL was 0.848 for predicting macroalbuminuria, with 70.6% sensitivity and 83.3% specificity at the cut-off value [33]; the findings are nearly consistent with the present AUC (0.816). A study reported the AUC of uNGAL reached 0.956 for normoalbuminuric DN [22], but the AUC was not comparable to the present AUC because of the different diagnostic objectives and study cohort. Although uCysC and uCLU are promising biomarkers for diabetic damage of renal tubules, the capability to diagnose DKD has rarely been reported. As shown in Figs. 2 and 3, both biomarkers had moderate accuracy in diagnosing DKD and macroalbuminuria.

The capability of a biomarker to diagnosis a renal insult early depends on whether or not the alteration in the level of the biomarker detected by current methods precedes the appearance of urinary microalbumin. With respect to being an early biomarker, Table 3 shows that the levels of uNGAL and uCysC were elevated in T2DM and DKD patients with a normal UACR (<30 mg/g•Cr). Thus, the alterations will be detected before the increase in urine microalbumin. Further, we consider that the possibilities of increased biomarkers in T2DM and DKD patients with a normal UACR are as follows: the biomarkers are specific for hyperglycemia without kidney injury; and/or the biomarkers are novel biomarkers for renal impairment and increase earlier than UACR. In the present study, however, the data shown in Table 4 indicates that the levels of biomarkers did not increase with the increase in HbA_1c_. Higher uNGAL and uCysC levels may not directly resulted from hyperglycemia, but from chronic tubular impairment, which may be more sensitive in the diagnosis of DKD than microalbuminuria, which is the current biomarker in use. Because biomarkers mirroring tubular impairment can be determined before microalbuminuria, biomarker levels predict the appearance of microalbumin in the urine. In previous studies, the AUC for uNGAL identifying microalbuminuria in T2DM patients was 0.759 with a sensitivity of 66.7% and a specificity of 88.9% [33]. Vikas Garg et al. [24] reported that once the values were corrected by urinary creatinine, the AUCs were promoted and achieved 0.956 (urinary NGAL-to-creatinine ratio) and 0.867 (urinary CysC-to-creatinine ratio). Our study showed that the AUCs reached 0.841 (uNGAL) and 0.805 (uCysC), which are high for clinical use and may be sensitive markers for early renal damage in T2DM; however, uCLU may not have the advantage of earlier elevation. The level of uCLU in the normal UACR group was similar to control subjects (Table 3), although the AUC (0.783) was significant for predicting microalbuminuria.

Previous investigations have demonstrated that NGAL is highly expressed in the kidney proximal tubules of diabetic rats [13, 26] and uNGAL is markedly elevated in microalbuminuric patients with T1DM [22, 34] or T2DM [35, 36], uNGAL is positively correlated with proteinuria, and can reflect the severity of diabetic nephropathy [36, 37]. Urinary CysC has also been suggested as an indicator of tubular dysfunction for the early diagnosis of DKD [21, 38, 39]. Previous results and conclusions include an early rise of uCysC in T2DM patients [24], an association with a declining GFR [21], and a rise in urine ACR [24], which are consistent with an alteration in the degree of albuminuria [21]. Compared with the preceding investigations, our study directly highlighted the clinical performance in the diagnosis of DKD and microalbuminuria and elucidated the clinical basis for use of early biomarkers.

As a biomarker for nephrotoxic lesions [16, 40], urinary CLU has not been investigated to identify the renal damage in clinical T2DM patients. We selected urinary CLU as a potential renal injury biomarker in T2DM patients for the following reasons. First, it has been confirmed that uCLU can reflect proximal tubular damage [41, 42], an important mechanism underlying diabetic renal insults. Second, CLU functions as a defense factor to prevent renal fibrosis [41, 43], the common nephropathogenesis of CKD. Third, the findings of animal experiments [25, 44] have demonstrated that the uCLU level is significantly elevated and closely associated with the severity of histopathologic nephropathy resulting from obesity. The present results showed that the level of uCLU markedly increased in renal-impaired patients, was positively correlated with albuminuria, and mirrored the severity of renal damage in the T2DM cohort. Moreover, uCLU has a suitable AUC with a reasonable sensitivity and specificity for the diagnosis of DKD, macroalbuminuria, and microalbuminuria, even though the elevation may not be earlier than microalbumin. We believe that the potential nephropathogenesis related to CLU is worthy of further study and the TGF-β-related hyperglycemia pathway may be the putative mechanism. High glucose stimulates TGF-β expression and bioactivity in the proximal tubule [45] via extracellular regulated kinase 1/2 and protein kinase C activation [30, 46] and promotes inflammatory cells, particularly T cells and macrophages, to release TGF-β, which are early events that set up fibrogenesis [47]. Highly-expressed TGF-β acts as the most potent cytokine for renal fibrogenesis and stimulus for epithelial-myofibroblast transdifferentiation of proximal tubular epithelial cells [30, 48]. Further, TGF-β induces the up-regulation of CLU in renal tubular epithelium via the activation of AP-1 transcriptor protein and protein kinase C [49]. By post-translation processing, CLU is immediately converted to two subtypes. The α subtype is excreted into the urine and the β subtype is accumulated in the cytoplasm of the renal tubular epithelial cells [50]. As a component of glomerular immune deposits, over-expressed CLU may be anti-apoptotic and pro-survival and attenuate the development of renal fibrosis [41].

The main limitation to our study was that it involved a relatively small research in a single center, thus needs further validation at the multi-center level as a consecutive cohort study.

## Conclusions

In conclusion, uNGAL and uCysC are promising biomarkers for the early diagnosis of DKD and prediction of microalbuminuria. Although uCLU could have a later than the onset of microalbuminuria, it has fair performance in distinguishing DKD from T2DM.

## Abbreviations

AKI: Acute kidney injury
AUCs: Areas under the receiver operating characteristic curve
CI: Confidence interval
CKD: Chronic kidney disease
CLU: Clusterin
CVs: Coefficients of variation
CysC: Cystatin C
DKD: Diabetic kidney disease
DN: Diabetic nephropathy
eGFR: Estimated glomerular filtration rate
HbA_1c_: Haemoglobin A_1C_
KDIGO: Kidney Disease Outcome Quality Initiative
NGAL: Urinary neutrophil gelatinase-associated lipocalin
T2DM: Type 2 diabetes mellitus
UACR: Urinary albumin creatinine ratio
uCLU: Urinary CLU
uCysC: Urinary CysC
uNGAL: Urinary NGAL
